# Effect of Huanglian Decoction on the Intestinal Microbiome in Stress Ulcer (SU) Mice

**DOI:** 10.1155/2021/3087270

**Published:** 2021-09-22

**Authors:** Qi Zhang, Jing-Jing Guo, Yuen-Ming Yau, Ying-Jie Wang, Yan-Bin Cheng, Xuan Tuo, Zong-Bao Yang, Lin-Chao Qian

**Affiliations:** ^1^Department of Traditional Chinese Medicine, School of Medicine, Xiamen University, Xiamen 361105, China; ^2^Department of Traditional Chinese Medicine, College of Medicine, Qinghai University, Xining 810000, China; ^3^School of Traditional Chinese Medicine, Xiamen University Malaysia, Sepang 43900, Malaysia

## Abstract

**Background:**

Stress ulcer (SU) is a serious gastrointestinal mucosal lesion under acute stress. Huanglian decoction is a famous traditional Chinese medicine prescription, which has been used to treat digestive system diseases for thousands of years. Many clinical cases have proved that Huanglian decoction has a good effect on SU. Some studies have shown that the intestinal bacteria will be changed accordingly when the gastrointestinal mucosa is damaged. However, there are few published reports on the effect of the intestinal microbiome with SU mice that were treated by Huanglian decoction. In this study, we investigated the effect of the fecal microbiome in mice with SU by the 16S rDNA sequencing technology.

**Methods:**

Male KM mice were induced by cold-restraint stress except for the normal control group and then treated by Huanglian decoction (Group HD) and Esomeprazole magnesium solution (Group ES) separately for 7 days. 16S rDNA sequencing technology analysis was applied to evaluate the changes of bacterial flora in mice feces. And, histopathological methods and molecular biological detection methods were also performed.

**Results:**

Huanglian decoction could help to repair the gastric mucosal injury and regulate the relative content of TNF-*α* and IL-1*β*. Moreover, Huanglian decoction could increase the relative abundance of intestinal probiotics in the intestine of mice with SU, especially in Verrucomicrobiae and *Akkermansia*.

**Conclusions:**

Huanglian decoction might effectively promote the repair of gastrointestinal mucosal injury and regulate the number and species of intestinal bacteria to maintain the stability of gastrointestinal microecology.

## 1. Introduction

Stress ulcer is an acute gastric mucosal lesion, which can be detected by gastroscope inflammatory erosion, superficial ulcer, and mucosal bleeding [1]. Its occurrence is quite urgent, often with abdominal pain, acid regurgitation, and even hematemesis and melena. If not effectively controlled, it is easy to lead to massive bleeding, or even death [2, 3]. In recent years, the incidence of stress ulcers is increasing [4, 5].

Intestinal microflora plays a significant role in the health of the host. Many pieces of research have already proved that changes of intestinal microflora are closely related to disease occurrence, especially gastrointestinal diseases in most cases [6–8]. It is reported that when the gastrointestinal tract is stimulated, the microecology of the flora will be out of balance, including the proportions of intestinal pathogenic bacteria increase and probiotics reduce [9, 10].

Huanglian decoction is a famous prescription of traditional Chinese medicine, which comes from Treatise on Febrile Diseases and is composed of Coptidis Rhizoma, Pinellia ternata, Zingiberis Siccatum Rhizoma, etc. In ancient times, it was usually used to treat stomachache, vomiting, and diarrhea. Pharmacological studies have shown that Huanglian decoction has an anti-inflammatory effect on our gastrointestinal system [11], but whether and how it would affect the intestinal microflora is rarely investigated.

In this study, on the stress ulcer model constructed with cold-restraint stress, the effects of Huanglian decoction on the intestinal microecology of SU mice were examined by 16s rDNA sequencing technology, aiming to provide a foundation for comprehensive utilization of Huanglian decoction.

## 2. Materials and Methods

### 2.1. Preparation of Huanglian Decoction

The traditional Chinese medicine (TCM) formula in our study was Huanglian decoction, composed of seven herbs, namely, Coptidis Rhizoma (“Huanglian” in Chinese), *Pinellia ternata* (“Banxia” in Chinese), Zingiberis Siccatum Rhizoma (“Ganjiang” in Chinese), *Codonopsis pilosula* (“Dangshen” in Chinese), Red Jujube (“Hongzao” in Chinese), Ramulus Cinnamomi (“Guizhi” in Chinese), and Radix Glycyrrhizae (“Gancao” in Chinese). Herbs were purchased from Kang Mei Pharmaceutical Co., Ltd. To ensure the quality of the Huanglian decoction used in the study, we had tested it through UHPLC-MS/MS method. It was indicated that the quality of Huanglian decoction was stable, of which many active components played a significant therapeutic effect for gastrointestinal diseases (Table S1).

We prepared Huanglian decoction following the methods of Ma [12]. Details are as follows: Coptidis Rhizoma (9 g), *Pinellia ternata* (12 g), Zingiberis Siccatum Rhizoma (9 g), *Codonopsis pilosula* (12 g), Red Jujube (30 g), Ramulus Cinnamomi (9 g), and Radix Glycyrrhizae (9 g) were soaked in 600 mL of distilled water for 1 h before being boiled for 30 min. Filtrates were collected, and the residues were then boiled with an addition of 300 mL water for 20 min. Finally, the two filtrates were combined and concentrated to a final volume of 54 mL (1.65 g/mL) by a rotary evaporation instrument (model: RE-52AA, Shanghai Yarong biochemical instrument factory). The filtrate was stored in a seal at 4°C.

### 2.2. Preparation of Esomeprazole Magnesium Solution

Esomeprazole magnesium enteric-coated tablet (20 mg) was ground into powder, added with 54 mL of distilled water to obtain Esomeprazole magnesium solution (0.37 mg/mL), and then stored at 4°C.

### 2.3. Animals

KM mice (SPF-grade, 6-weak-old male, 25–30 g on average) were purchased from Beijing Huafukang Biotechnology Co., Ltd. (Beijing, China, license number: SCXK 2019-0008). All mice were housed in a temperature-controlled room (24°C∼26°C) of the Medical College of Xiamen University with a 12-hour light/dark cycle. The experiment had been approved by the Animal Ethics Committee of Xiamen University (Permit Number: XMULAC20190142), and the procedures were strictly in accordance with the guidance on treating experimental animals issued by the Ministry of Science and Technology of The People's Republic of China. Mice were acclimatized to the laboratory for 7 days prior to the experiments. Subsequently, all mice were randomly divided into the normal control group (NC), stress ulcer group (SU), Huanglian decoction group (HD), and Esomeprazole magnesium solution group (ES), and each group consisted of 6 mice.

### 2.4. Construction of the Stress Ulcer Model

Mice were deprived of food for 24 hours prior to the model construction and all groups except for the NC were established. The SU model was established by cold-restraint stress [13]: mice were restrained by fixing the four limbs to a wooden board using strings and placed in a thermostatic water tank at 20°C for 10 hours, of which the horizontal surface was submerged into the breastbone of mice. After successful molding, the obvious ulcer focal could be seen, which indicated that the SU model had been successfully prepared.

### 2.5. Treatment Processes

After successful modeling, the Group HD was treated with orally administered Huanglian decoction (0.1 ml/10 g) and Group ES with Esomeprazole magnesium solution (0.1 ml/10 g) for 7 days, while the NC Group and SU Group were treated with 0.9% NaCl solution. All groups were sacrificed by cervical dislocation at Day 16, and the feces, gastric, and duodenal tissues were collected under sterile conditions and preserved at −80°C.

### 2.6. Evaluation of the Gastric Ulcer Index

The gastric tissue of mice was cut from pylorus to cardia along the great curvature of the stomach, and the residues in the stomach were washed with normal saline. Then, it was unfolded to fully expose the ulcer. The area of gastric ulcer was measured by an observer using a vernier caliper that was unaware of the experimental regimen. The score was calculated according to the standard of Guth et al. [14]: 1: a small spot erosion; 2: the ulceration length <1 mm; 3: the ulceration length 1-2 mm; 4: the ulceration length 2-3 mm; and 5: the ulceration length >3 mm. The score was doubled when the ulceration width was >1 mm.

### 2.7. Histopathology

The gastric tissue of 0.5 cm × 0.5 cm was collected and washed with sterile 0.9% NaCl solution. The samples were fixed in 4% formaldehyde solution, dehydrated with gradient ethanol, and sectioned with 5 *μ*m thickness after paraffin embedding. The histopathological changes of gastric mucosa were observed under a light microscope after hematoxylin and eosin staining. At the same time, pathological images of the gastric mucosa of mice were captured by an image acquisition system.

### 2.8. ELISA Analysis

The duodenal mucosa (0.5 cm × 0.5 cm) of mice was collected for ELISA analysis. The duodenal mucosa of each group was cut up and homogenized with 200 *μ*L PBS and then centrifuged at 4°C and 12000 rpm for 20 min. The supernatant was extracted, and standard samples with different concentrations were prepared, added into the standard well, and mixed gently. After incubation, enzyme addition, and washing, 90 *μ*L substrate solution (TMB) was added to develop color, and termination solution was added to terminate the reaction. The absorbance (OD value) of each well was measured at 450 nm wavelength, and the content of each tissue index (1 g/ml) was calculated according to the standard curve.

### 2.9. Analysis of Fecal 16S rRNA

The composition of fecal microbiota was detected by 16S rRNA sequencing analysis. Microbial genomic DNA of fecal flora was extracted from the fecal samples of mice in each group, and the concentration, purity, and integrity of DNA were determined by Multiskan™ GO Thermo Fisher Scientific (USA) and agarose gel electrophoresis.

DNA from each fecal sample was amplified at the V3-V4 region of the 16S rRNA genes by using specific primers: 341F and 806R. Then, the library was constructed and quantified by Qubit 3.0, Agilent 2100, and Q-PCR. Finally, PE250 sequencing of Illumina HiSeq2500 (Illumina, San Diego, CA, USA) was performed. The nonrepetitive sequences were clustered by OTU based on the Clean Tage. According to the clustering results, Alpha, Beta diversity analyses were performed and the composition of intestinal microflora at different species levels was analyzed.

### 2.10. Statistical Analysis

Data were statistically processed by GraphPad Prism 7.0 and SPSS 21 software. Measurement data were expressed as mean ± standard deviation $\left(\bar{x}\pm s\right)$. Both the statistical method of one-way ANOVA and the Student–Newman–Keuls (SNK) method were used for multiple comparisons among the groups. The *P* values <0.05 indicated that the difference was statistically significant.

## 3. Results

### 3.1. Weight Observation of Mice

**Figure 1:**
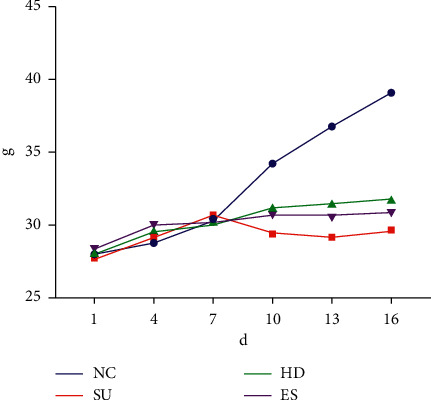
Changes of mice weight during experiment. The horizontal axis indicates the experimental days, and the vertical axis indicates the weight of mice. The lines of different colors indicate the weight changes of different groups of samples.

The first seven days of the experiment was the adaptation period of the mice, so the weight of the mice increased steadily. After modeling, compared with the NC group, the weight of the SU Group decreased significantly (*P* < 0.05), which supported our successful modeling. At the same time, the weight growth of the HD and ES group was slower than the NC Group, but there was no significant difference among the three groups (Figure 1).

### 3.2. Behavioural Change of Mice

Comparing with the NC group, mice in Group SU had mental depression with less hair cleanliness, decreased diet, and reduced activity, which gathered in the corner of the rat cage. After medication, mice showed a better mental state with a sensitive response and high activity. Meanwhile, they had better hair quality, and their diet mostly returned to normal.

### 3.3. Gastric Ulcer Index Detection

There is a great increase in the ulcer index after modeling. The index of the gastric ulcer was significantly reduced in Group HD and Group ES comparing with the SU Group, but there is no significant difference between the two treatment groups (Table S2).

### 3.4. Observation of Histomorphology and Histopathology

After mice were molded by cold-restraint stress, gastric tissue swelling, mucosal congestion, bleeding points, blood clots, and linear bleeding zone could be seen by the naked eyes in the SU Group, which proved that the SU model was established successfully (Figure S1).

**Figure 2:**
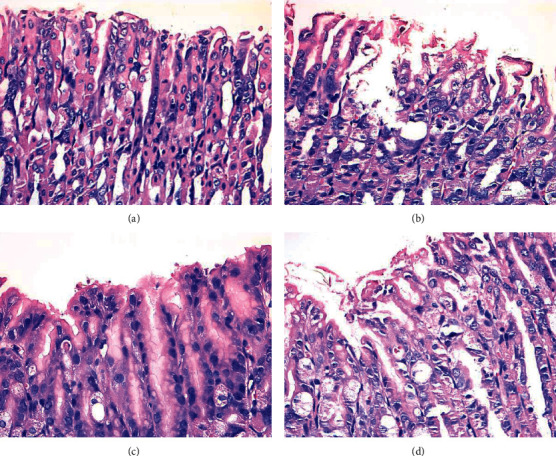
Histological morphology of gastric mucosa from 4 groups. (a) Mice in Group NC; (b) mice in Group SU; (c) mice in Group HD; (d) mice in Group ES. Scale bars represent 50 *μ*m in each group.

In Group NC, the structure of gastric mucosa and muscle layer was intact; the morphology of epithelial cells and glands was normal and orderly; and no inflammatory cell infiltration, congestion, edema, and shedding were found. On the contrary, the gastric mucosa in the Group SU was damaged obviously, whose arrangement was disordered, and the local gland cells were broken, fallen off, and sunken to form an ulcer. Compared with Group SU, the structure of gastric mucosa and muscle layer in the Group HD was basically returned to normal, and the cells arranged orderly. In the Group ES, the surface of gastric mucosa was partially shedding, some glandular cells arranged orderly, which was close to the normal arrangement but had not yet been restored (Figure 2).

### 3.5. ELISA Analysis

**Figure 3:**
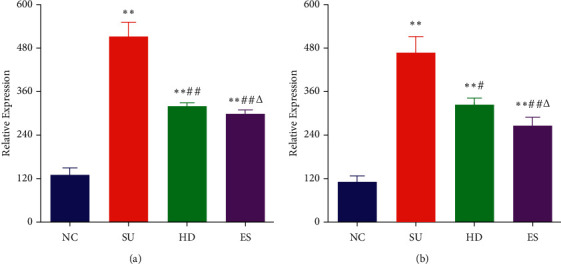
The expression of TNF-*α* (a) and IL-1*β* (b) in duodenal mucosa in mice of each group. *∗*vs. NC, *P* < 0.05; *∗∗*vs. NC, *P* < 0.01; #vs. SU, *P* < 0.05; ##vs. SU, *P* < 0.01; Δvs. HD, *P* > 0.05.

The relative expressions of TNF-*α* (Figure 3(a)) and IL-1*β* (Figure 3(b)) in the duodenal mucosa of SU mice were detected by ELISA. These two indexes in the duodenal mucosa of Group SU showed higher expression than that in NC. After treatment with Huanglian decoction and Esomeprazole magnesium solution respectively, the expressions of TNF-*α* and IL-1*β* decreased significantly in Groups HD and ES. However, there was no significant difference in the relative content of TNF-*α* and IL-1*β* between Group HD and Group ES.

### 3.6. Diversity Analysis of Intestinal Microflora

**Figure 4:**
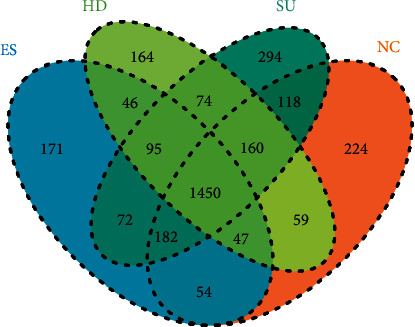
Venn diagram of OTUs. Each circle of different colors represents different groups. The number of overlapped parts within the circle indicates common OTUs among the groups, and the number outside the overlapping parts indicates the unique OTUs in each sample group.

The OTU clustering Venn diagram could analyze the common and unique OTUs between different groups, which was plotted using the OTU abundance table. Groups NC, SU, HD, and ES had 1450 common OTUs and 224, 294, 164, and 17l unique OTUs, respectively, (Figure 4).

### 3.7. Variations in Alpha Diversity

Alpha diversity was used to reflect the abundance (ACE, Chaol, observed species), diversity (Shannon and Simpson), and evenness (*J*) of the microbial community. From the results of this experiment, we found that every Alpha diversity index of the last two treatment groups was much lower than that of the NC Group (all *P* < 0.01). On the contrary, the SU Group showed an opposite trend at every index of Alpha diversity compared with Groups HD and ES, which significantly increased after model construction. It might be inferred that the intestinal flora of mice would change differently after modeling and treatments, which was also the basis of our study (Table 1).

**Figure 5:**
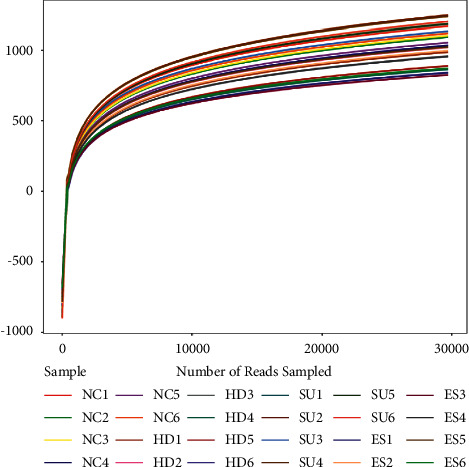
The dilution curve of the sample. The horizontal axis represents the different sequencing depths of the sample, and the vertical axis represents the dilution index at the corresponding depth. The curves of different colors represent the dilution curves of different samples. The curve increases until it is smooth, indicating that the sequencing depth is enough to cover most of the microorganisms in the sample.

**Figure 6:**
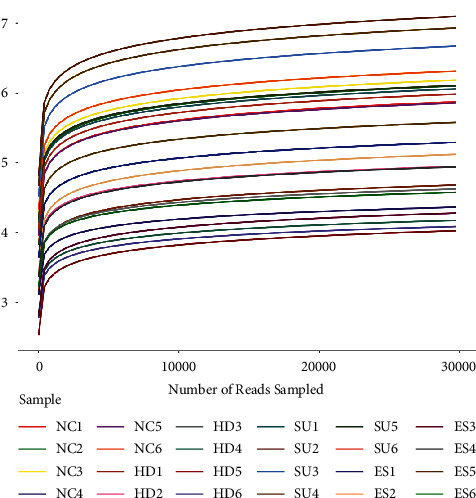
The Shannon index curve. The horizontal axis represents the different sequencing depths of the sample, and the vertical axis represents the Shannon index at the corresponding depth. The curves of different colors represent the Shannon curves of different samples. The curve increases until it is smooth, indicating that the sequencing depth is enough to cover most of the microorganisms in the sample.

Meanwhile, there are two curves cited in the essay to prove the sufficiency and rationality of data. As shown in the figures, the dilution curve (Figure 5) and the Shannon Wiener curve (Figure 6) both tend to be flat, indicating the amount of sequencing data is reasonable, and more data has little contribution to the discovery of new OTUs.

### 3.8. Characterization of Microbiota

**Figure 7:**
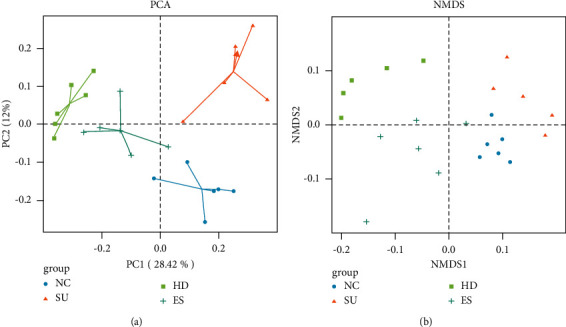
PCA analysis (a) and NMDS analysis (b). Different colors in the graph represent different groups of samples. A point is a sample. The closer the points are, the more similar the samples are, otherwise, the greater the difference is.

PCA analysis and NMDS analysis were used to visualize differences in intestinal microbial composition across the four groups. Based on the results of PCA analysis in the four groups, it was found that their flora was clustered within each group, which had great dispersion and significant differences (Figure 7(a)). As is shown in NMDS plots, the difference among groups was larger than that within groups, indicating that the four groups of bacteria were comparable (Figure 7(b)).

### 3.9. Analysis of Fecal Microbiota at the Phylum and Family Levels

To know more about the connection between microbial diversity and therapy, we coevaluated the intestinal flora of different mice by 16S rDNA gene sequences, studied the microbial structure at phylum and family level primarily, and generated the column accumulation map of relative microbial abundance through the first 20 microbial species of each sample. It is shown that the relative abundance of intestinal flora after treatment varied dramatically at the phylum and family level.

**Figure 8:**
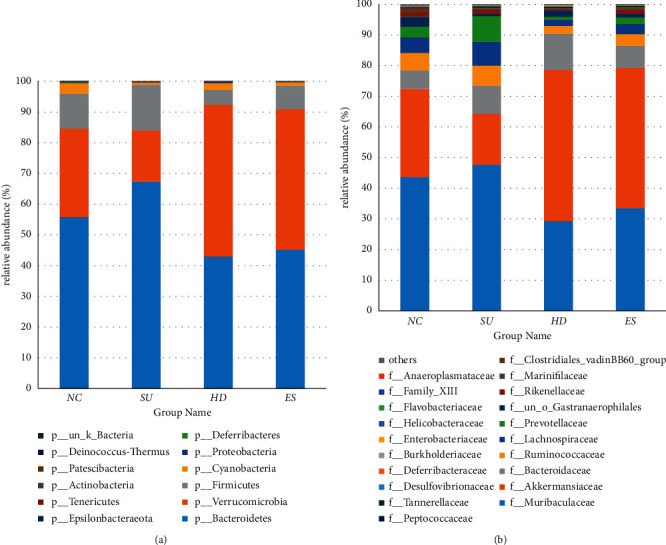
Accumulation map of mice intestinal microorganism abundance at the phylum and family level. The horizontal coordinate represents different groups, and the ordinate represents relative abundance; different colors represent different microbial species. (a) Relative abundance of the top 20 dominant species at the phylum level. (b) Relative abundance of the top 20 dominant species at the family level.

Bacteroidetes, Firmicutes, and Verrucomicrobia were the predominant strains in mice at the phylum level (Figure 8(a)). In Group SU, over 67% in average proportion was Bacteroidetes, which was higher than Group NC (55.67%), Group ES (45.15%), and Group HD (42.95%), and over 14% was Firmicutes (higher than 11.44% in Group NC, 7.53% in Group ES, and 4.87% in Group HD). On the contrary, Verrucomicrobia in Group SU (16.63% in average proportion) was less than that in Group NC (28.81%). However, the proportion of Verrucomicrobia ascended distinctly in Group HD (49.43%) and Group ES (45.78%) after treating with Huanglian decoction and Esomeprazole magnesium solution.

Muribaculaceae, Akkermansiacease, Bacteroidaceae, Lachnospiraceae, Ruminococcaceae, Prevotellaceae, etc. accounted for a substantial part of strains at the family level. Muribaculaceae was the amplest in all groups (Figure 8(b)). The abundance of Muribaculaceae in Groups HD and ES was 29.21% and 33.51%, which were lower than Group NC (43.59%) and Group SU (47.65%). As one of the families in Verrucomicrobia, the abundance of Akkermansiaceae was in the second place, which decreased after successful SU model establishment, while increasing significantly after treatments had been given. According to the above analysis, we found that the flora in the intestine of mice would change differently after cold-restraint stress and treatments.

### 3.10. Difference Analysis of Intestinal Microflora

**Figure 9:**
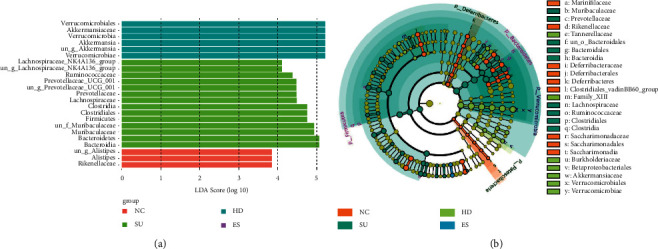
Difference analysis of mice intestinal microflora. (a) LEfSe analysis between the four groups of mice, bar graph lengths representing LDA values; (b) evolutionary branch plot analysis, showing species with significant differences in abundance between the four groups.

LDA Effect Size (LEfSe) analysis can realize the comparison between multiple groups to find the species with a significant difference in abundance between groups and assess the impact on them. From our final results, we noticed that the number of different OTU units in abundance was 3, 14, and 6 in Groups NC, SU, and HD, respectively, (Figure 9(a)). Most notably, Bacteroidetes in Group SU and Verrucomicrobia and Akkermansiaceae in Group HD scored above 5, which indicated that these significantly different species had a great impact.

Furthermore, from the LEfSe-based evolutionary branch diagram (Figure 9(b)), we could clearly see the different compositions of intestinal bacteria in each group. Marinifilaceae, Rikenellaceae, Deferribacteraceae, Deferribacterales, Deferribacteres, Clostridiales_vadinBB60_group, Saccharimonadaceae, Saccharimonadales, and Saccharimonadia were more abundant in Group NC. Muribaculaceae, Prevotellaceae, un_o_Bacteroidales, Bacteroidales, Bacteroidia, Lachnospiraceae, Ruminococcaceae, Clostridiales, and Clostridia were more abundant in Group SU. Tannerellaceae, Family_XIII, Burkholderiaceae, Betaproteobacteriales, Akkermansiaceae, Verrucomicrobiales, and Verrucomicrobiae were more abundant in Group HD.

## 4. Discussion

There are many stressors of SU, which can be triggered by severe trauma, critical illness, strong mental and psychological pressure, and some drugs or food with strong stimulation to the stomach [15]. In this experiment, the cold-restraint stress was used to establish the model, in which the mice were fixed in the cold and water immersion environment to simulate the stress ulcer [16]. This modeling method has been widely used in animal models of acute digestive tract mucosal injury [17].

In recent years, the incidence of SU has been increasing due to the improvement of productivity and unbalanced diet [18]. At present, the mean treatments available for SU are drugs such as antacids, proton pump inhibitors, H2 receptor blockers, sucralfate, or surgical treatment, of which prevention is the best but least noticed [19]. Growing concerns have been raised regarding whether the long-term use of western medicine will lead to side effects [20]. Traditional Chinese medicine, with its unique advantages, has gradually played an irreplaceable role in the prevention and treatment of SU.

SU belongs to the category of “stomachache” in traditional Chinese medicine. It is mainly caused by improper diet, emotional disorder, and frailty, which can lead to the disorder of yin and yang, the deficiency of spleen and stomach, stagnant movement of Qi and blood, and finally mucosal erosion.

Huanglian decoction comes from article 173 of Zhong-jing Zhang's “Treatise on Febrile Diseases.” It is a famous prescription for the treatment of digestive system diseases. Clinical research has shown that based on dialectical accuracy, Huanglian decoction can effectively treat many gastrointestinal diseases, such as gastric ulcer, duodenal ulcer, and stress ulcer. It has obtained a curative effect on improving symptoms such as stomachache, belching, and fatigue significantly [21, 22]. Moreover, another study has shown that Huanglian decoction also has a good therapeutic effect on *Helicobacter pylori* complicated gastric ulcer [23]. Pharmacological studies have proved that many single herbs in Huanglian decoction can play an effective role in anti-inflammatory, bactericidal, and gastric mucosal protection [24–30].

Ulceration is related to the decrease of the protective function of gastric mucosa, the increase of proinflammatory factors and inflammatory mediators, and the imbalance of gastric mucosal barrier function [31]. Therefore, regulation of inflammatory factors might be an effective way to treat SU.

In this experiment, after the successful establishment of the model, the pathological damage of gastric mucosa was detected under histopathological observation, and the duodenal inflammatory factors TNF-*α* and IL-1*β* were detected by ELISA. It was indicated that the gastric mucosa got severely injured due to SU and the two duodenal inflammatory factors were significantly increased. On the contrary, compared with the SU Group, both Huanglian decoction and Esomeprazole magnesium could effectively repair the gastric mucosal injury and inhibit the uncontrolled release of TNF-*α* and IL-1*β* after an intragastric intervention. It was therefore concluded that Huanglian decoction might be able to adjust the level of inflammatory factors and mobilize the immune system of the body.

As one of the initial pathological factors of SU, dysbacteriosis occupies an important position [32]. In the intestinal microecology of a normal human body, the flora can make the corresponding self-regulation according to the changes of the external environment within a certain range, so as to maintain a relatively balanced state. When the external stimulus factors exceed the acceptable range, the flora microenvironment will be out of balance, resulting in changes in the structure, type, and quantity of the flora, followed by a great occurrence of diseases [33–35].

In this experiment, the results of *α* diversity, PCA and NMDS analysis, and the composition of flora structure showed that the inflammation caused by the SU model changed the proportion of dominant bacteria and inferior bacteria in the intestinal flora, and the inferior bacteria were obviously transformed into dominant bacteria, resulting in many adverse effects. After being treated with Huanglian decoction, this change could be adjusted to increase the species and proportion of beneficial bacteria and reduce the pathogenic bacteria.

In the NC group, the highest three relative expression dominant groups were Rikenellaceae, *Alistipes*, and un_g_*Alistipes*. Studies have shown that if Rikenellaceae is reduced, which maintains the normal state of the intestinal immune system [36], it will often affect the immune function of the human body and result in obesity and other diseases [37]. *Alistipes* is a relatively new genus of bacteria within the Bacteroidetes phylum. In a healthy body, their presence is correlated with the promotion of healthy phenotypes to protect the body from diseases such as colitis or cardiovascular fibrotic disorders [38]. They may also play a leading role in the development of the diseases. Therefore, they serve as both beneficial bacteria and Opportunistic pathogen in the normal specimen intestinal tract to maintain a normal physiological state of the intestinal tract together.

In Group SU, the highest three relative expression dominant groups were Bacteroidia, Bacteroidetes, and Bacteroidales. Bacteroidetes not only are considered as the leading defenders of the healthy immune system inhabiting our gastrointestinal tract [39] but also play specific roles in the acceleration of pathological inflammation, immune dysregulation, and gastrointestinal disorders [40]. The SU specimen showed that Bacteroides proliferated rapidly and became the dominant bacteria group after the success of modeling, which destroyed the gastrointestinal mucosa. Meanwhile, research has shown that Firmicutes/Bacteroidetes ratio is positively correlated with bowel health [41], though it was found that the ratio of F/B decreased in the SU Group, indicating that the SU model harmed gut microbiota. Furthermore, it was also observed that Muribaculaceae had increased, a beneficial bacterium [42], which can inhibit the production of pathogenic bacteria due to its self-repair function.

The highest three relative expression dominant groups in the HD group were Verrucomicrobiae, un_g_*Akkermansia*, and *Akkermansia*. Verrucomicrobia is a Gram-negative bacterium, and *Akkermansia* is a mucin-degrading bacterium of the phylum Verrucomicrobia, which is involved in maintaining intestinal integrity and is considered to be a promising candidate as probiotics [43, 44]. It has been known that *Akkermansia* can reduce inflammation, protect gut health, and play a key role in improving the host metabolic functions and immune responses [44–46]. Notably, increased levels of *Akkermansia* and Verrucomicrobia were observed after giving Huanglian decoction, which suggested it might have potential anti-inflammatory properties by regulating the abundance of beneficial bacteria and achieve a therapeutic effect.

According to the above, beneficial bacteria dominated and maintained a healthy intestinal tract of mice in the NC group. When the SU model was successfully established, the proportion of opportunistic bacteria in the Group SU increased rapidly and became the dominant bacteria. It showed a proinflammatory response and promoted the process of gastrointestinal pathological changes, leading to stress ulcer. After treatments, the number and types of beneficial bacteria increased and became the dominant bacteria again to eliminate the intestinal inflammatory reaction, especially in Group HD.

## 5. Conclusion

Huanglian decoction has a positive effect on SU mice, similar to Esomeprazole magnesium solution. It could repair the acute gastric mucosal injury caused by SU, regulate the imbalance expression of TNF-*α* and IL-1*β* on duodenal mucosa, and reduce the inflammatory reaction of the gastrointestinal tract under stress. More importantly, it could increase the number of beneficial bacteria and promote the balance of gastrointestinal microecology by regulating the type, proportion, and quantity of intestinal flora.

It is indicated that the imbalance of flora might be an important reason for intestinal inflammation, and the changes of inflammatory factors in turn lead to the deterioration of unbalanced intestinal microecology. Huanglian decoction may act as a defender to break the vicious circle, which plays an anti-inflammatory role in the SU mice by restoring the species and quantity of flora, in order to repair gastrointestinal mucosa and improve gastrointestinal discomfort.

In conclusion, this study laid a foundation for a better understanding of the intestinal microbiome changes during the treatment of SU with Huanglian decoction. Yet, the mechanism of how Huanglian decoction alters intestinal flora needs to be elucidated in future studies.

## Figures and Tables

**Table 1:** The OTU Alpha diversity index of intestinal microflora $\left(\bar{x}\pm s\right)$.

Group	Observe	Chao1	ACE	Shannon	Simpson
NC	1224.83 ± 45.23	1679.85 ± 49.61	1712.59 ± 44.44	4.02 ± 0.24	0.9 ± 0.04
SU	1330.33 ± 43.34	1775.81 ± 47.46	1835.09 ± 44.43∗	4.39 ± 0.32	0.94 ± 0.03
HD	1052 ± 72.81^*∗∗#*^	1489.52 ± 78.3^*∗∗#*^	1553.45 ± 57.31^*∗∗#*^	3 ± 0.26^*∗∗#*^	0.75 ± 0.05^*∗∗#*^
ES	1040 ± 91.75^*∗∗#*Δ^	1442.25 ± 101.75^*∗∗#*Δ^	1509.07 ± 107.82^*∗∗#*Δ^	3.26 ± 0.34^*∗∗#*Δ^	0.78 ± 0.06^*∗∗#*Δ^

Data presented as mean ± standard error of the mean. *∗*vs. NC, *P* < 0.05; *∗∗*vs. NC, *P* < 0.01; #vs. SU, *P* < 0.05; ##vs. SU, *P* < 0.01; Δvs. HD, *P* > 0.05.

## Data Availability

All the original data used to support the findings of this study were supplied by Zong-Bao Yang under license and so cannot be provided free of charge. Requests for access to these data should be made to the corresponding author Zong-Bao Yang, yangzb@xmu.edu.cn.

## Supplementary Materials

Supplementary Materials Figure S1: comparison of gastric mucosal morphology between the (a) NC Group and (b) SU Group of mice. Table S1: parameters of UHPLC-MS/MS conditions for active components of Huanglian decoction. Table S2: data are presented as mean ± standard error of the mean. (*∗*: vs. NC *P* < 0.05; *∗∗*: vs. NC *P* < 0.01; #: vs. SU *P* < 0.05; Δ: vs. HD *P* > 0.05). ().

## References

[1] Haglund U. (1990). Stress ulcers. *Scandinavian Journal of Gastroenterology*.

[2] Elsaed W. M., Alahmadi A. M., Al-Ahmadi B. T., Taha J. A., Tarabishi R. M. (2018). Gastroprotective and antioxidant effects of fluvoxamine on stress-induced peptic ulcer in rats. *Journal of Taibah University Medical Sciences*.

[3] Butterfield W. C. (1975). Experimental stress ulcers: a review. *Surgery Annual*.

[4] Thakur E. R., Sansgiry S., Kramer J. R. (2020). The incidence and prevalence of anxiety, depression, and post-traumatic stress disorder in a national cohort of US veterans with inflammatory bowel disease. *Inflammatory Bowel Diseases*.

[5] Cui G., Yuan A. (2018). A systematic review of epidemiology and risk factors associated with Chinese inflammatory bowel disease. *Frontiers of Medicine*.

[6] Franzosa E. A., Sirota-Madi A., Avila-Pacheco J. (2019). Gut microbiome structure and metabolic activity in inflammatory bowel disease. *Nature Microbiology*.

[7] Hills R. D., Pontefract B. A., Mishcon H. R., Black C. A., Sutton S. C., Theberge C. R (2019). Gut microbiome: profound implications for diet and disease. *Nutrients*.

[8] Nishida A., Inoue R., Inatomi O., Bamba S., Naito Y., Andoh A. (2018). Gut microbiota in the pathogenesis of inflammatory bowel disease. *Clinical Journal of Gastroenterology*.

[9] Kim S.-K., Guevarra R. B., Kim Y.-T. (2019). Role of probiotics in human gut microbiome-associated diseases. *Journal of Microbiology and Biotechnology*.

[10] Sanders M. E., Merenstein D. J., Reid G., Gibson G. R., Rastall R. A. (2019). Probiotics and prebiotics in intestinal health and disease: from biology to the clinic. *Nature Reviews Gastroenterology & Hepatology*.

[11] Qin C. L., Liu J. Y., Cheng Z. M. (1994). Pharmacological studies on the effects of Huanglian decoction on experimental gastric lesions in rats and antiemetic in pigeons. *Zhongguo Zhongyao Zazhi*.

[12] Ma S. P. (2015). *Experimental for Pharmacology of Traditional Chinese Medicine*.

[13] Wang F.-Y., Liu J. M., Luo H. H., Liu A. H., Jiang Y. (2015). Potential protective effects of *Clostridium butyricumon* experimental gastric ulcers in mice. *World Journal of Gastroenterology*.

[14] Guth P. H., Aures D., Paulsen G. (1979). Topical aspirin plus HCl gastric lesions in the rat. *Gastroenterology*.

[15] Murison R., Olafsen K. (1991). Stress ulceration in rats: impact of prior stress experience. *Neuroscience & Biobehavioral Reviews*.

[16] Goodman A. A., Osborne M. P. (1972). An experimental model and clinical definition of stress ulceration. *Surgery Gynecology & Obstetrics*.

[17] Ephgrave K. S., Cullen J. J., Broadhurst K., Kleiman‐Wexler R., Shirazi S. S., Schulze‐Delrieu K. (1997). Gastric contractions, secretions and injury in cold restraint. *Neuro-Gastroenterology and Motility*.

[18] Tan B., Norman R., Litton E. (2016). Incidence and cost of stress ulcer prophylaxis after discharge from the intensive care unit: a retrospective study. *Critical care and Resuscitation: Journal of the Australasian Academy of Critical Care Medicine*.

[19] Schiessel R., Feil W., Wenzl E. (1990). Mechanisms of stress ulceration and implications for treatment. *Gastroenterology Clinics of North America*.

[20] Jaynes M., Kumar A. B. (2019). The risks of long-term use of proton pump inhibitors: a critical review. *Therapeutic advances in drug safety*.

[21] Lin Y. Q. (2006). Therapeutic effect of supplemented Huanglian decoction on 50 cases of peptic ulcer. *Guangxi Medical Journal*.

[22] Cai B. (2004). Therapeutic effect of supplemented Huanglian decoction on 50 cases of peptic ulcer. *New Chinese Medicine*.

[23] Zhou J. R. (2015). Effect of Huanglian decoction combined with western medicine on *Helicobacter pylori* infection complicated with gastric ulcer. *Journal of Practical Traditional Chinese Medicine*.

[24] Endo M., Hori M., Mihara T. (2017). Zingiberis Siccatum Rhizoma, the active component of the Kampo formula Daikenchuto, induces anti-inflammatory actions through *α*7 nicotinic acetylcholine receptor activation. *Neuro-Gastroenterology and Motility: The Official Journal of the European Gastrointestinal Motility Society*.

[25] Fujii A., Okuyama T., Wakame K., Okumura T., Ikeya Y., Nishizawa M. (2017). Identification of anti-inflammatory constituents in Phellodendri Cortex and Coptidis Rhizoma by monitoring the suppression of nitric oxide production. *Journal of Natural Medicines*.

[26] Lin Y.-C., Chang C.-W., Wu C.-R. (2015). Anti-nociceptive, anti-inflammatory and toxicological evaluation of Fang-Ji-Huang-Qi-Tang in rodents. *BMC Complementary and Alternative Medicine*.

[27] Liu J., Zhang Q., Li R.-L. (2020). The traditional uses, phytochemistry, pharmacology and toxicology of *Cinnamomi ramulus*: a review. *Journal of Pharmacy and Pharmacology*.

[28] Meng Y., Xu Y., Chang C. (2020). Extraction, characterization and anti-inflammatory activities of an inulin-type fructan from *Codonopsis pilosula*. *International Journal of Biological Macromolecules*.

[29] Tang D., Yan R., Sun Y., Kai G., Chen K., Li J. (2020). Material basis, effect, and mechanism of ethanol extract of Pinellia ternata tubers on oxidative stress-induced cell senescence. *Phytomedicine*.

[30] Zou K., Li Z., Zhang Y. (2017). Advances in the study of berberine and its derivatives: a focus on anti-inflammatory and anti-tumor effects in the digestive system. *Acta Pharmacologica Sinica*.

[31] Kim H., Banerjee N., Sirven M. A. (2017). Pomegranate polyphenolics reduce inflammation and ulceration in intestinal colitis-involvement of the miR-145/p70S6K1/HIF1*α* axis *in vivo* and *in vitro*. *The Journal of Nutritional Biochemistry*.

[32] Xue T., Wang L. J., Wu Y. Q. (2020). Effect of acupuncture on serum inflammatory cytokines and intestinal flora in rats with stress-induced gastric ulcer. *Zhen Ci Yan Jiu*.

[33] Labanski A., Langhorst J., Engler H., Elsenbruch S. (2020). Stress and the brain-gut axis in functional and chronic-inflammatory gastrointestinal diseases: a transdisciplinary challenge. *Psychoneuroendocrinology*.

[34] Richard M. L., Sokol H. (2019). The gut *mycobiota*: insights into analysis, environmental interactions and role in gastrointestinal diseases. *Nature Reviews Gastroenterology & Hepatology*.

[35] Sidhu M., Van der Poorten D. (2017). The gut microbiome. *Australian Family Physician*.

[36] Schanz O., Chijiiwa R., Cengiz S. C. (2020). Dietary AhR ligands regulate AhRR expression in intestinal immune cells and intestinal microbiota composition. *International Journal of Molecular Sciences*.

[37] Arnoriaga-Rodríguez M., Mayneris-Perxachs J., Burokas A. (2020). Gut bacterial ClpB-like gene function is associated with decreased body weight and a characteristic microbiota profile. *Microbiome*.

[38] Parker B. J., Wearsch P. A., Veloo A. C. M., Rodriguez-Palacios A. (2020). The genus Alistipes: gut bacteria with emerging implications to inflammation, cancer, and mental health. *Frontiers in Immunology*.

[39] Ley R. E., Turnbaugh P. J., Klein S., Gordon J. I. (2006). Human gut microbes associated with obesity. *Nature*.

[40] Wexler H. M. (2007). Bacteroides: the good, the bad, and the nitty-gritty. *Clinical Microbiology Reviews*.

[41] Stojanov S., Berlec A., Štrukelj B. (2020). The influence of probiotics on the firmicutes/bacteroidetes ratio in the treatment of obesity and inflammatory bowel disease. *Microorganisms*.

[42] Sibai M., Altuntaş E., Yıldırım B., Öztürk G., Yıldırım S., Demircan T. (2020). Microbiome and longevity: high abundance of longevity-linked *Muribaculaceae* in the gut of the long-living rodent spalax leucodon. *OMICS: A Journal of Integrative Biology*.

[43] Geerlings S. Y., Kostopoulos I., De Vos W. M., Belzer C. (2018). Akkermansia muciniphila in the human gastrointestinal tract: when, where, and how?. *Microorganisms*.

[44] Zhang T., Li Q., Cheng L., Buch H., Zhang F. (2019). Akkermansia muciniphila is a promising probiotic. *Microbial Biotechnology*.

[45] Zhai Q., Feng S., Arjan N., Chen W. (2019). A next generation probiotic, *Akkermansia muciniphila*. *Critical Reviews in Food Science and Nutrition*.

[46] Derrien M., Belzer C., De Vos W. M. (2017). *Akkermansia muciniphila* and its role in regulating host functions. *Microbial Pathogenesis*.

