# Supplementary material for: Effect of Huanglian Decoction on the Intestinal Microbiome in Stress Ulcer (SU) Mice
Source: Evid Based Complement Alternat Med. 2021 Sep 22;2021:3087270. doi: 10.1155/2021/3087270 (PMC8483906; doi:10.1155/2021/3087270)
Supplement: Supplementary Materials — Figure S1: comparison of gastric mucosal morphology between the (a) NC Group and (b) SU Group of mice. Table S1: parameters of UHPLC-MS/MS conditions for active components of Huanglian decoction. Table S2: data are presented as mean ± standard error of the mean. (∗: vs. NC P < 0.05; ∗∗: vs. NC P < 0.01; #: vs. SU P < 0.05; Δ: vs. HD P > 0.05). (). [file 3087270.f1.zip › 3087270.f1/Fig S1 (1).docx]

Fig S1


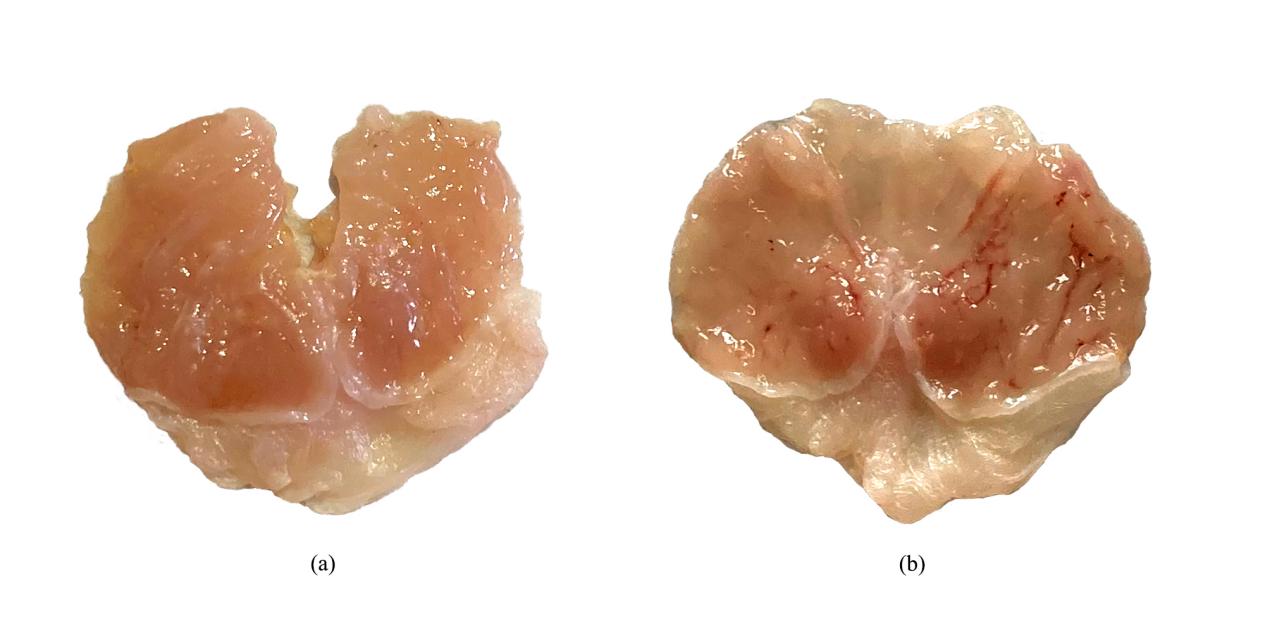
(a) (b)

Figure S1: Comparison of gastric mucosal morphology between the (a) NC group and (b) SU group of mice
