# Supplementary material for: Effect of Huanglian Decoction on the Intestinal Microbiome in Stress Ulcer (SU) Mice
Source: Evid Based Complement Alternat Med. 2021 Sep 22;2021:3087270. doi: 10.1155/2021/3087270 (PMC8483906; doi:10.1155/2021/3087270)
Supplement: Supplementary Materials — Figure S1: comparison of gastric mucosal morphology between the (a) NC Group and (b) SU Group of mice. Table S1: parameters of UHPLC-MS/MS conditions for active components of Huanglian decoction. Table S2: data are presented as mean ± standard error of the mean. (∗: vs. NC P < 0.05; ∗∗: vs. NC P < 0.01; #: vs. SU P < 0.05; Δ: vs. HD P > 0.05). (). [file 3087270.f1.zip › 3087270.f1/Supplementary Material.docx]

**Effect of Huanglian decoction on the intestinal microbiome in stress ulcer (SU) mice**

Qi Zhang^1^, Jing-jing Guo^1^, Yuen-ming Yau^1^, Ying-jie Wang^2^, Yan-bin Cheng^1^, Xuan Tuo^1^, Zong-bao Yang^1*^, Lin-chao Qian^1,3*^

^*^Correspondence to: Prof. Lin-chao Qian ([lcqian@xmu.edu.cn](mailto:lcqian@xmu.edu.cn)); Prof. Zong-bao Yang [(yangzb@xmu.edu.cn)](mailto:(yangzb@xmu.edu.cn);)

^1^ Department of Traditional Chinese Medicine, School of Medicine, Xiamen University, Xiamen 361105, China

^2^ Department of Traditional Chinese Medicine, College of Medicine, Qinghai University, Xining 810000, China

^3^ School of Traditional Chinese Medicine, Xiamen University Malaysia, Sepang 43900, Malaysia

**Observation of Gastric Mucosal Morphology**

The gastric tissue of mice was cut from pylorus to cardia along the great curvature of the stomach, and the residues in the stomach were washed with normal saline. As shown in Figure 1, after mice were molded by cold-restraint stress, gastric tissue swelling, mucosal congestion, bleeding points, blood clots, and linear bleeding zone could be seen by naked eyes in the SU group, which proved that the SU model was established successfully. (Fig. S1)


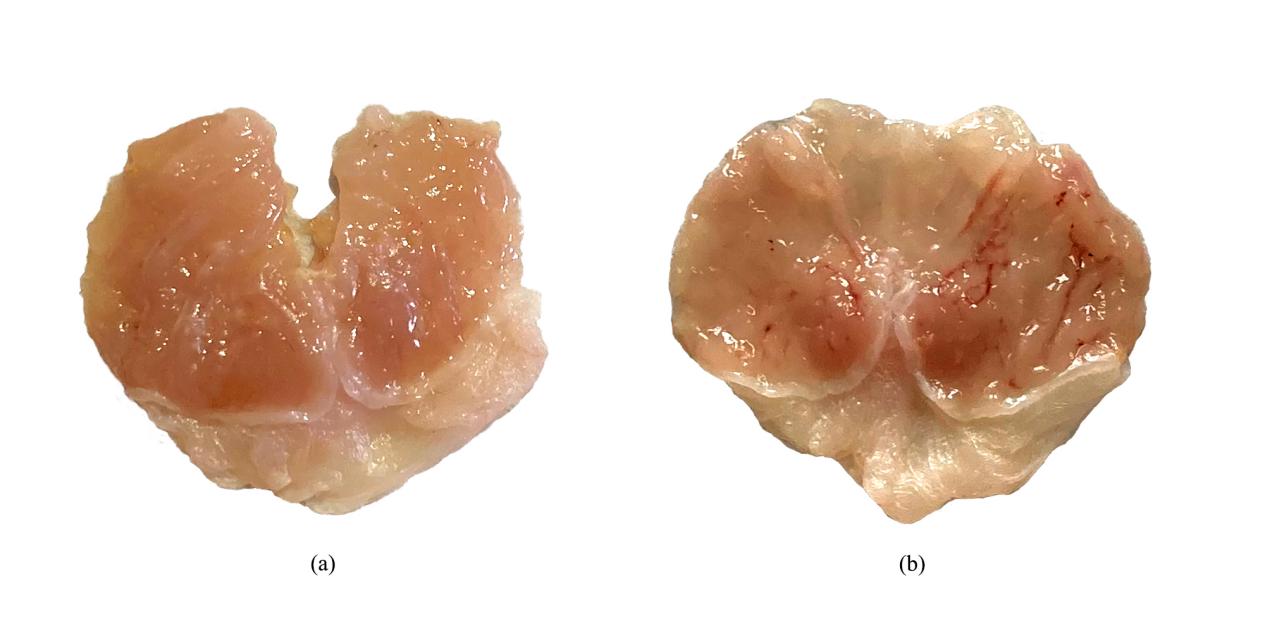


**Figure S1**: Comparison of gastric mucosal morphology between the (a) NC group and (b) SU group of mice

**Analysis of Huanglian decoction UHPLC-MS/MS**

In this study, an ultra-high-performance liquid chromatography-tandem mass spectrometry (UHPLC-MS/MS) method combined with a high-resolution electric spray ionization mass (HR-ESI-MS) detector were used to simultaneously determine multiple bioactive compounds in Huanglian decoction. The analysis was performed on Cosmosil CN column (2.6μm, 250 mm×4.6 mm, 5μm, Nakalai Tesque Co., Ltd, Kyoto Japan) with a mobile phase consisting of acetonitrile and 0.1% formic acid at a flow rate of 1.0 mL/min with gradient elution. The column temperature was 30 ℃. The detection wavelength was set at 254 nm, and the injection volume was 5 μL. The high-resolution ESI-MS detector was used to record the UHPLC chromatography and was analyzed on the Thermo Q-Exactive system. The results are shown in Table S1.

According to UHPLC-MS/MS analysis, there were 55 active compounds, including Coptisine, Evodin, Berberine, Epiberberine, Columbamine, Magnoflorine, Obacunone, Obaculactone in Coptidis Rhizoma, Cinnamaldehyde, Coumarin, Cinnamyl acetate, β-Cadinene in Ramulus Cinnamomi，and Umbelliferone, Liquiritigenin, Glycyrrhizin, Isoliquiritin, Liquiritin in Radix Glycyrrhizae. Meanwhile, there were 15 active components, Gingerol，Protocatechualdehyde, 2-Undecanone, Asparagic acid, Pentaldehyde oxime, 3-Methyleicosane, 2-Methylpyrazine, Daucosterin, Citral, Shogaol, 2-Aminobutanoic acid, Baicalin, Ephedrine, Ethyl palmitate, Methyl-2-Chloropropenoate in Pinellia Pernata, and Serine, L-Proline, L-Arginine, Tyrosine, Taraxerol, Glutamic acid, Tangshenoside, Phenylalanine, Isoleucine, Aspartic acid in Codonopsis Pilosula. Moreover, β-Phellandrene, 1,8-Cineole, Zingiberene, 6-Shogaol, 6-Gingerdione, Gingerenone A, and Hexahydrocurcumin were detected in Zingiberis Siccatum Rhizoma, and Betulinic acid, Malic acid, Stepharine, N-Nornuciferine, Asimilobine, Thiamine were found in Red Jujube.

To sum up, the quality of Huanglian decoction was stable, of which many active components played a significant therapeutic effect for gastrointestinal diseases.

| **Table S1** Parameters of UHPLC-MS/MS conditions for active components of Huanglian decoction | | | | | | | |
| --- | --- | --- | --- | --- | --- | --- | --- |
| Common name | Chinese name | Component | Molecular weight | Molecular formula | ESI ion value | Retention time of single drug (min) | Retention time in Huanglian decoction (min) |
| Coptidis Rhizoma | Huang Lian | Coptisine | 321.3262 | C19H14NO4(+) | 322.326 | 27.07 | 27.07 |
|  |  | Evodin | 329.3472 | C18H19NO5 | 330.347 | 2.53 | 2.49 |
|  |  | Berberine | 336.3607 | C20H18NO4(+) | 337.361 | 2.95 | 3.14 |
|  |  | Epiberberine | 336.3607 | C20H18NO4 | 337.3607 | 2.95 | 3.14 |
|  |  | Columbamine | 338.3766 | C20H20NO4(+) | 339.377 | 7.29 | 7.27 |
|  |  | Magnoflorine | 342.4083 | C20H24NO4(+) | 341.4083 | 2.77 | 2.79 |
|  |  | Obacunone | 454.519 | C26H30O7 | 455.519 | 8.35 | 8.35 |
|  |  | Obaculactone | 470.518 | C26H30O8 | 471.518 | 7.75 | 7.74 |
| Ramulus Cinnamomi | Gui Zhi | Cinnamaldehyde | 132.1592 | C9H8O | 155.1592 | 20.86 | 20.85 |
|  |  | Coumarin | 148.1353 | C9H6O2 | 147.1353 | 25.24 | 25.25 |
|  |  | Cinnamyl acetate | 176.2118 | C11H12O2 | 175.2118 | 15.79 | 15.72 |
|  |  | β-Cadinene | 204.355 | C15H24 | 203.355 | 11.56 | 11.42 |
| Radix Glycyrrhizae | Gan Cao | Umbelliferone | 162.14 | C9H6O3 | 163.14 | 9.35 | 9.35 |
|  |  | Liquiritigenin | 256.25338 | C15H12O4 | 257.25338 | 11.81 | 11.81 |
|  |  | Glycyrrhizin | 327.37434 | C19H21NO4 | 328.37434 | 5.59 | 5.58 |
|  |  | Isoliquiritin | 418.39 | C21H22O9 | 419.39 | 14.66 | 14.68 |
|  |  | Liquiritin | 822.93 | C42H62O16 | 823.93 | 12.74 | 12.67 |
| Pinellia Pernata | Ban Xia | Gingerol | 350.4923 | C21H34O4 | 351.4923 | 2.36 | 2.55 |
|  |  | Protocatechualdehyde | 138.1207 | C7H6O3 | 139.1207 | 2.32 | 2.34 |
|  |  | 2-Undecanone | 170.2918 | C11H22O | 171.2918 | 16.09 | 16.02 |
|  |  | Asparagic acid | 133.1 | C4H7NO4 | 134.1 | 2.28 | 2.34 |
|  |  | Pentaldehyde oxime | 101.15 | C5H11NO | 102.15 | 2.34 | 2.34 |
|  |  | 3-Methyleicosane | 94.1145 | C5H6N2 | 117.1145 | 2.3 | 2.7 |
|  |  | 2-Methylpyrazine | 576.8473 | C35H60O6 | 599.8473 | 8.64 | 8.59 |
|  |  | Daucosterin | 112.2126 | C8H16 | 135.2126 | 2.3 | 2.28 |
|  |  | Citral | 152.2334 | C10H16O | 175.2334 | 2.57 | 2.76 |
|  |  | Shogaol | 276.3707 | C17H24O3 | 277.3707 | 23.18 | 23.15 |
|  |  | 2-Aminobutanoic acid | 102.1118 | C4H9NO2 | 103.1118 | 2.34 | 2.30 |
|  |  | Baicalin | 446.361 | C21H18O11 | 447.361 | 2.32 | 2.30 |
|  |  | Ephedrine | 167.2247 | C10H15NO | 168.2247 | 2.34 | 2.53 |
|  |  | Ethyl palmitate | 286.4896 | C18H36O2 | 287.4896 | 2.38 | 2.36 |
|  |  | Methyl-2-Chloropropenoate | 123.5528 | C4H5ClO2 | 124.5528 | 2.26 | 2.34 |
| Codonopsis Pilosula | Dang Shen | Serine | 105.09 | C3H7NO3 | 106.09 | 2.79 | 2.83 |
|  |  | L-Proline | 115.1305 | C5H9NO2 | 116.1305 | 2.81 | 2.7 |
|  |  | L-Arginine | 174.2 | C6H14N4O2 | 175.2 | 2.56 | 2.59 |
|  |  | Tyrosine | 181.189 | C9H11NO3 | 180.189 | 3.53 | 3.71 |
|  |  | Taraxerol | 426.7 | C30H50O | 449.7 | 23.1 | 23.06 |
|  |  | Glutamic acid | 147.13076 | C5H9NO4 | 148.13076 | 2.39 | 2.34 |
|  |  | Tangshenoside | 678.6 | C29H42O18 | 677.6 | 7.53 | 7.71 |
|  |  | Phenylalanine | 165.19 | C9H11NO2 | 164.19 | 5.25 | 5.32 |
|  |  | Isoleucine | 131.17 | C6H13NO2 | 130.17 | 3.67 | 3.71 |
|  |  | Aspartic acid | 133.1 | C4H7NO4 | 172.1 | 3.4 | 3.43 |
| Zingiberis Siccatum  Rhizoma | Gan Jiang | β-Phellandrene | 136.23 | C10H16 | 175.23 | 2.49 | 2.45 |
|  |  | 1,8-Cineole | 154.25 | C10H18O | 177.25 | 23.33 | 23.33 |
|  |  | Zingiberene | 204.35 | C15H24 | 205.35 | 6.78 | 6.77 |
|  |  | 6-Shogaol | 276.4 | C17H24O3 | 277.4 | 23.33 | 23.31 |
|  |  | 6-Gingerdione | 292.4 | C17H24O4 | 291.4 | 23.28 | 23.3 |
|  |  | Gingerenone A | 356.4 | C21H24O5 | 357.4 | 17.74 | 17.73 |
|  |  | Hexahydrocurcumin | 374.4 | C21H26O6 | 373.4 | 17.75 | 17.74 |
| Red Jujube | Hong Zao | Betulinic acid | 456.7 | C30H48O3 | 455.7 | 2.95 | 2.85 |
|  |  | Malic acid | 134.09 | C4H6O5 | 133.09 | 2.47 | 2.43 |
|  |  | Stepharine | 297.3 | C18H19NO3 | 320.3 | 2.4 | 4.45 |
|  |  | N-Nornuciferine | 281.3 | C18H19NO2 | 304.3 | 2.56 | 2.57 |
|  |  | Asimilobine | 267.32 | C17H17NO2 | 268.32 | 5.69 | 5.74 |
|  |  | Thiamine | 300.81 | C12H17ClN4OS | 339.81 | 2.35 | 2.57 |

**Gastric Ulcer Index Detection**

There is a great increase in the ulcer index after modeling. The index of gastric ulcer was significantly reduced in Group HD and ES comparing with the SU group, but there is no significant difference between the two treatment groups. (Table. S2)

| **Table S2: Gastric ulcer index** | | | | |
| --- | --- | --- | --- | --- |
| Group |  |  |  | Ulcer index |
| NC |  |  |  | 0.00±0.00^△^ |
| SU |  |  |  | 16.83±5.81^**^ |
| HD |  |  |  | 2.33±2.07^#^ |
| ES |  |  |  | 3.17±2.93^#△^ |

**Table S2**: Data are presented as mean ± standard error of the mean.

*: Vs NC P＜0.05; **: Vs NC P＜0.01; #: Vs SU P＜0.05; △: Vs HD P＞0.05
