# Supplementary material for: Effect of Huanglian Decoction on the Intestinal Microbiome in Stress Ulcer (SU) Mice
Source: Evid Based Complement Alternat Med. 2021 Sep 22;2021:3087270. doi: 10.1155/2021/3087270 (PMC8483906; doi:10.1155/2021/3087270)
Supplement: Supplementary Materials — Figure S1: comparison of gastric mucosal morphology between the (a) NC Group and (b) SU Group of mice. Table S1: parameters of UHPLC-MS/MS conditions for active components of Huanglian decoction. Table S2: data are presented as mean ± standard error of the mean. (∗: vs. NC P < 0.05; ∗∗: vs. NC P < 0.01; #: vs. SU P < 0.05; Δ: vs. HD P > 0.05). (). [file 3087270.f1.zip › 3087270.f1/Table S1 (1).docx]

| **Table S1** Parameters of UHPLC-MS/MS conditions for active components of Huanglian decoction | | | | | | | |
| --- | --- | --- | --- | --- | --- | --- | --- |
| Common name | Chinese name | Component | Molecular weight | Molecular formula | ESI ion value | Retention time of single drug (min) | Retention time in Huanglian decoction (min) |
| Coptidis Rhizoma | Huang Lian | Coptisine | 321.3262 | C19H14NO4(+) | 322.326 | 27.07 | 27.07 |
|  |  | Evodin | 329.3472 | C18H19NO5 | 330.347 | 2.53 | 2.49 |
|  |  | Berberine | 336.3607 | C20H18NO4(+) | 337.361 | 2.95 | 3.14 |
|  |  | Epiberberine | 336.3607 | C20H18NO4 | 337.3607 | 2.95 | 3.14 |
|  |  | Columbamine | 338.3766 | C20H20NO4(+) | 339.377 | 7.29 | 7.27 |
|  |  | Magnoflorine | 342.4083 | C20H24NO4(+) | 341.4083 | 2.77 | 2.79 |
|  |  | Obacunone | 454.519 | C26H30O7 | 455.519 | 8.35 | 8.35 |
|  |  | Obaculactone | 470.518 | C26H30O8 | 471.518 | 7.75 | 7.74 |
| Ramulus Cinnamomi | Gui Zhi | Cinnamaldehyde | 132.1592 | C9H8O | 155.1592 | 20.86 | 20.85 |
|  |  | Coumarin | 148.1353 | C9H6O2 | 147.1353 | 25.24 | 25.25 |
|  |  | Cinnamyl acetate | 176.2118 | C11H12O2 | 175.2118 | 15.79 | 15.72 |
|  |  | β-Cadinene | 204.355 | C15H24 | 203.355 | 11.56 | 11.42 |
| Radix Glycyrrhizae | Gan Cao | Umbelliferone | 162.14 | C9H6O3 | 163.14 | 9.35 | 9.35 |
|  |  | Liquiritigenin | 256.25338 | C15H12O4 | 257.25338 | 11.81 | 11.81 |
|  |  | Glycyrrhizin | 327.37434 | C19H21NO4 | 328.37434 | 5.59 | 5.58 |
|  |  | Isoliquiritin | 418.39 | C21H22O9 | 419.39 | 14.66 | 14.68 |
|  |  | Liquiritin | 822.93 | C42H62O16 | 823.93 | 12.74 | 12.67 |
| Pinellia Pernata | Ban Xia | Gingerol | 350.4923 | C21H34O4 | 351.4923 | 2.36 | 2.55 |
|  |  | Protocatechualdehyde | 138.1207 | C7H6O3 | 139.1207 | 2.32 | 2.34 |
|  |  | 2-Undecanone | 170.2918 | C11H22O | 171.2918 | 16.09 | 16.02 |
|  |  | Asparagic acid | 133.1 | C4H7NO4 | 134.1 | 2.28 | 2.34 |
|  |  | Pentaldehyde oxime | 101.15 | C5H11NO | 102.15 | 2.34 | 2.34 |
|  |  | 3-Methyleicosane | 94.1145 | C5H6N2 | 117.1145 | 2.3 | 2.7 |
|  |  | 2-Methylpyrazine | 576.8473 | C35H60O6 | 599.8473 | 8.64 | 8.59 |
|  |  | Daucosterin | 112.2126 | C8H16 | 135.2126 | 2.3 | 2.28 |
|  |  | Citral | 152.2334 | C10H16O | 175.2334 | 2.57 | 2.76 |
|  |  | Shogaol | 276.3707 | C17H24O3 | 277.3707 | 23.18 | 23.15 |
|  |  | 2-Aminobutanoic acid | 102.1118 | C4H9NO2 | 103.1118 | 2.34 | 2.30 |
|  |  | Baicalin | 446.361 | C21H18O11 | 447.361 | 2.32 | 2.30 |
|  |  | Ephedrine | 167.2247 | C10H15NO | 168.2247 | 2.34 | 2.53 |
|  |  | Ethyl palmitate | 286.4896 | C18H36O2 | 287.4896 | 2.38 | 2.36 |
|  |  | Methyl-2-Chloropropenoate | 123.5528 | C4H5ClO2 | 124.5528 | 2.26 | 2.34 |
| Codonopsis Pilosula | Dang Shen | Serine | 105.09 | C3H7NO3 | 106.09 | 2.79 | 2.83 |
|  |  | L-Proline | 115.1305 | C5H9NO2 | 116.1305 | 2.81 | 2.7 |
|  |  | L-Arginine | 174.2 | C6H14N4O2 | 175.2 | 2.56 | 2.59 |
|  |  | Tyrosine | 181.189 | C9H11NO3 | 180.189 | 3.53 | 3.71 |
|  |  | Taraxerol | 426.7 | C30H50O | 449.7 | 23.1 | 23.06 |
|  |  | Glutamic acid | 147.13076 | C5H9NO4 | 148.13076 | 2.39 | 2.34 |
|  |  | Tangshenoside | 678.6 | C29H42O18 | 677.6 | 7.53 | 7.71 |
|  |  | Phenylalanine | 165.19 | C9H11NO2 | 164.19 | 5.25 | 5.32 |
|  |  | Isoleucine | 131.17 | C6H13NO2 | 130.17 | 3.67 | 3.71 |
|  |  | Aspartic acid | 133.1 | C4H7NO4 | 172.1 | 3.4 | 3.43 |
| Zingiberis Siccatum  Rhizoma | Gan Jiang | β-Phellandrene | 136.23 | C10H16 | 175.23 | 2.49 | 2.45 |
|  |  | 1,8-Cineole | 154.25 | C10H18O | 177.25 | 23.33 | 23.33 |
|  |  | Zingiberene | 204.35 | C15H24 | 205.35 | 6.78 | 6.77 |
|  |  | 6-Shogaol | 276.4 | C17H24O3 | 277.4 | 23.33 | 23.31 |
|  |  | 6-Gingerdione | 292.4 | C17H24O4 | 291.4 | 23.28 | 23.3 |
|  |  | Gingerenone A | 356.4 | C21H24O5 | 357.4 | 17.74 | 17.73 |
|  |  | Hexahydrocurcumin | 374.4 | C21H26O6 | 373.4 | 17.75 | 17.74 |
| Red Jujube | Hong Zao | Betulinic acid | 456.7 | C30H48O3 | 455.7 | 2.95 | 2.85 |
|  |  | Malic acid | 134.09 | C4H6O5 | 133.09 | 2.47 | 2.43 |
|  |  | Stepharine | 297.3 | C18H19NO3 | 320.3 | 2.4 | 4.45 |
|  |  | N-Nornuciferine | 281.3 | C18H19NO2 | 304.3 | 2.56 | 2.57 |
|  |  | Asimilobine | 267.32 | C17H17NO2 | 268.32 | 5.69 | 5.74 |
|  |  | Thiamine | 300.81 | C12H17ClN4OS | 339.81 | 2.35 | 2.57 |
