# Supplementary material for: Effect of Huanglian Decoction on the Intestinal Microbiome in Stress Ulcer (SU) Mice
Source: Evid Based Complement Alternat Med. 2021 Sep 22;2021:3087270. doi: 10.1155/2021/3087270 (PMC8483906; doi:10.1155/2021/3087270)
Supplement: Supplementary Materials — Figure S1: comparison of gastric mucosal morphology between the (a) NC Group and (b) SU Group of mice. Table S1: parameters of UHPLC-MS/MS conditions for active components of Huanglian decoction. Table S2: data are presented as mean ± standard error of the mean. (∗: vs. NC P < 0.05; ∗∗: vs. NC P < 0.01; #: vs. SU P < 0.05; Δ: vs. HD P > 0.05). (). [file 3087270.f1.zip › 3087270.f1/Table S2 (1).docx]

| **Table S2: Gastric ulcer index** | | | | |
| --- | --- | --- | --- | --- |
| Group |  |  |  | Ulcer index |
| NC |  |  |  | 0.00±0.00^△^ |
| SU |  |  |  | 16.83±5.81^**^ |
| HD |  |  |  | 2.33±2.07^#^ |
| ES |  |  |  | 3.17±2.93^#△^ |

Table S2: Data are presented as mean ± standard error of the mean.

*: Vs NC P＜0.05; **: Vs NC P＜0.01; #: Vs SU P＜0.05; △: Vs HD P＞0.05
